# Antimicrobial Resistance in Nontyphoidal *Salmonella* and Clinically Relevant *Enterococcus* From Faecal Samples of Conservation‐Priority Captive Ungulates in a United Arab Emirates Urban Zoo: A Cross‐Sectional Baseline Study

**DOI:** 10.1111/1758-2229.70416

**Published:** 2026-09-16

**Authors:** Ihab Habib, Moneeb A. Qablan, Fatema Aaref Alzaabi, Fatema Rashed Alnuaimi, Ghalya Ahmed Almemari, Glindya Bhagya Lakshmi, Safeya Al Naqbi, Fatima Al Shamsi, Dhanya Vijay, John Mhongovoyo, Sultan Khalifa Alkaabi, Amna Mana Alotaiba

**Affiliations:** ^1^ Department of Veterinary Medicine, College of Agriculture and Veterinary Medicine United Arab Emirates University Al Ain UAE; ^2^ ASPIRE Research Institute for Food Security in the Drylands (ARIFSID), United Arab Emirates University Al Ain UAE; ^3^ Al‐Ain Zoo Al Ain UAE

**Keywords:** antimicrobial resistance, environmental surveillance, one health, *Salmonella enterica*, zoological institutions

## Abstract

Antimicrobial resistance (AMR) is a One Health challenge driven by microbial exchange among humans, animals and the environment. Zoological institutions offer useful settings for environmental AMR surveillance. This single‐zoo cross‐sectional study examined the occurrence, antimicrobial susceptibility and genomic characteristics of nontyphoidal *Salmonella enterica* (NTS) and clinically relevant *Enterococcus* spp. in faecal samples from 101 clinically healthy captive ungulates representing seven conservation‐priority species at a major urban zoo in the United Arab Emirates. NTS was detected in 4/101 samples (3.9%), including serovars Schwarzengrund (*n* = 2), Kentucky (*n* = 1) and Chester (*n* = 1). Among the four recovered NTS isolates, all met the study MDR definition within the tested panel, including a *Salmonella* Kentucky ST198 isolate carrying multiple resistance genes and quinolone‐associated mutations. *Enterococcus* spp. were detected in 77/101 samples (76.2%), dominated by *Enterococcus faecium* and *Enterococcus casseliflavus* (each 41.5%). Among 33 *E. faecium/Enterococcus faecalis* isolates tested phenotypically, resistance was generally low, with erythromycin and ciprofloxacin resistance each observed in 9.1%. One clinically important *E. faecium* isolate showed glycopeptide resistance and genetic markers associated with reduced daptomycin susceptibility. These single‐institution cross‐sectional data provide an initial regional baseline for AMR‐relevant enteric bacteria in conservation‐managed ungulates and identify priorities for broader longitudinal and interface‐based surveillance.

## Graphical abstract

Targeted faecal surveillance of conservation‐priority captive ungulates identified multidrug‐resistant nontyphoidal *Salmonella* and clinically relevant *Enterococcus* in a UAE urban zoo. These findings provide cross‐sectional baseline evidence supporting cautious integration of zoo‐based monitoring into One Health antimicrobial resistance surveillance.

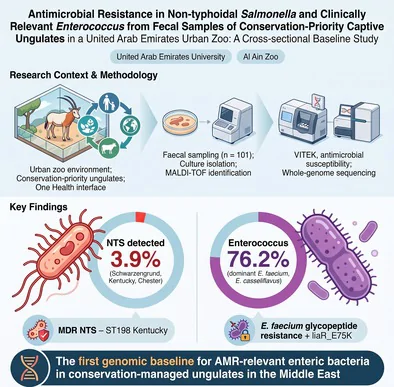

## 1 Introduction

Antimicrobial resistance (AMR) is widely recognised as an environmental as well as a public health problem, shaped by microbial exchange across connected humans, animals and built ecosystems (Larsson et al. 2023). Zoos may contribute to One Health‐oriented AMR surveillance because they bring diverse animal populations into managed settings near staff, visitors and urban environments (Caramujo et al. 2026). However, faecal surveys alone can only indicate carriage in sampled animals and cannot by themselves resolve transmission across the human–animal–environment interface (Nishigaki et al. 2025).

Among enteric bacteria of One Health concern, nontyphoidal *Salmonella enterica* (NTS) remains a leading cause of gastroenteritis globally and is also an important pathogen for animals (Galán‐Relaño et al. 2023). In managed collections, NTS has been associated with outbreaks in captive populations and has been documented as an infection concern in children's petting zoo settings, reflecting the potential for close contact environments to facilitate exposure (Friedman et al. 1998; Göttling et al. 2022). From an AMR perspective, NTS is also a priority because resistant strains can compromise treatment of invasive infections in vulnerable human populations and complicate veterinary care in valuable captive animals (Marin et al. 2022). Whole‐genome sequencing (WGS) has become central to modern *Salmonella* surveillance because it enables high‐resolution typing and detection of resistance determinants (Galán‐Relaño et al. 2023). In zoological collections, this genomic lens can help distinguish sporadic introductions from persistent circulation within enclosures or shared management systems, thereby supporting targeted interventions that protect animal health and reduce occupational exposure risks for keepers and veterinary staff (Stenger et al. 2025).

In parallel, *Enterococcus* spp. provide a complementary indicator for AMR monitoring across One Health systems (Zaidi et al. 2024). Enterococci are widespread in faeces and the environment, tolerate diverse stressors and readily acquire and disseminate resistance traits, making them valuable sentinels of antimicrobial selection pressure in humans, animals and environmental compartments (Zaidi et al. 2024). While zoonotic transmission pathways for resistant enterococci are complex and context‐dependent, their detection in managed animal populations is operationally important for stewardship (choice of empiric therapy) and environmental hygiene within zoo settings (Kim et al. 2024).

Zoological institutions in the UAE play key roles in *ex situ* conservation, veterinary care and reintroduction‐oriented programmes for conservation‐priority native ungulate species such as the Arabian oryx (Chanyandura et al. 2025). Because conservation‐managed ungulates may be moved between facilities for breeding, veterinary interventions or collection management, systematic screening for zoonotic enteric bacteria and AMR can yield benefits that support animal care, biosecurity and evidence‐based husbandry (Habib et al. 2026). In urban zoos, these benefits are amplified by the proximity of staff and visitors and by the broader environmental connectivity inherent to city settings (Kleespies et al. 2022). Despite the strategic value of such screening for enteric bacteria and their AMR, there remains limited baseline information on NTS and *Enterococcus* spp. circulating in zoo‐housed, native conservation‐priority ungulates in the UAE and the wider region.

Therefore, in this single‐institution cross‐sectional study we investigated faecal carriage, species/serovar composition and phenotypic AST profiles of NTS and clinically relevant *Enterococcus* in conservation‐priority captive ungulates. WGS was performed for all recovered NTS isolates and for one phenotypically prioritised *Enterococcus faecium* isolate. This study highlights the value of integrated phenotypic and genomic approaches for detecting clinically relevant resistance and provides a pilot baseline to inform zoo biosecurity and One Health surveillance in conservation‐managed ungulates.

## 2 Materials and Methods

### 2.1 Study Site, Animals and Faecal Sampling

Sampling was undertaken at Al Ain Zoo (https://www.alainzoo.com/; Al Ain City, eastern UAE), a large urban zoological institution that maintains extensive native and exotic collections and supports regional conservation and captive‐breeding programmes (Chanyandura et al. 2025). Study animals were clinically healthy captive ungulates selected through a husbandry‐integrated convenience sampling aligned with daily management schedules and welfare considerations so that procedures could be completed without restraint or disturbance to the animals.

In this study we relied exclusively on freshly voided faeces collected noninvasively from enclosure floors during routine husbandry, with no animal handling beyond standard care. Accordingly, formal animal ethics approval was not required for this component of the study. This design was intended to maximise welfare‐compatible sampling coverage, but it was not a random population survey; therefore, prevalence estimates and host‐species comparisons should be interpreted descriptively. Collection was performed by trained zookeepers during morning rounds. To ensure correct attribution to the defecating individual, samples were taken immediately after observed defecation with the animal visually confirmed at the time of collection. Disposable gloves and sterile single‐use tools were used for each sample, and faeces were transferred into individually labelled sterile containers to reduce the risk of environmental or between‐sample contamination (Habib et al. 2026).

In total, 101 individual ungulates were sampled from an overall source population of approximately 250 animals across the targeted species held at the zoo during the study period. The 101 sampled animals represented approximately 40% of the target source population, but selection depended on animal availability, observed defecation and keeper access during the sampling window. The sampled animals comprised Arabian oryx ( *Oryx leucoryx* ) (*n* = 18; 17.9%), scimitar‐horned oryx ( *Oryx dammah* ) (*n* = 18; 17.9%), addax ( *Addax nasomaculatus* ) (*n* = 21; 20.8%), common eland ( *Taurotragus oryx* ) (*n* = 16; 15.8%), Mhorr (dama) gazelle ( *Nanger dama mhorr* ) (*n* = 16; 15.8%), dama (Dana) gazelle ( *Nanger dama* ) (*n* = 9; 8.9%) and beisa oryx ( *Oryx beisa* ) (*n* = 3; 2.9%). This sample set represented the largest feasible number of individuals that could be included during the sampling window while maintaining broad coverage of conservation‐priority ungulate taxa in the collection. The sampling window was conducted over 3 months (September–November 2025). After collection, specimens were held at 4°C and transported on the day of sampling to the Veterinary Public Health Research Laboratory, United Arab Emirates University, for bacteriological testing. All samples were processed within 24 h of collection.

### 2.2 Isolation and Confirmation of NTS and Enterococcus spp.

Faecal samples (~5 g) from each animal were aseptically transferred into 45 mL of buffered peptone water (BPW; Oxoid, UK) (1:9 w/v) and homogenised. The cultures were incubated at 37°C for 18–24 h for nonselective pre‐enrichment (Sodagari et al. 2020). For *Salmonella* spp. isolation, the BPW pre‐enrichment cultures were further processed according to standard specifies detection of *Salmonella* spp. in environmental samples, animal feed and human consumption (ISO (International Organization for Standardization) 2002). After 36 h of pre‐enrichment at 37°C, aliquots (1 mL) were inoculated into Muller–Kauffmann tetrathionate novobiocin (TTN) broth (Neogen, USA) and incubated at 37°C for 48 h. In parallel, 0.1 mL of the pre‐enrichment was inoculated into modified semisolid Rappaport–Vassiliadis (MSRV) medium (Neogen, USA) and incubated at 41.5°C for 48 h. MSRV plates were examined at 24 h for characteristic *Salmonella* migration (a turbid white zone with radius > 10 mm); plates without visible growth were incubated an additional 24 h. After selective enrichment, aliquots were streaked onto xylose lysine deoxycholate (XLD) agar (Oxoid, UK) and incubated at 37°C for 24 h (ISO (International Organization for Standardization) 2002). Colonies displaying typical *Salmonella* morphology (e.g., red colonies with black centres on XLD) were picked (one isolate per sample was identified) and purified by subculturing on nutrient agar (37°C, 24 h) (Sodagari et al. 2020).

For *Enterococcus* spp. isolation, we used direct plating procedures (Habib et al. 2022). Briefly, the homogenate (1:9 w/v) of faecal samples (10^−1^) underwent further serial dilutions (10^−2^–10^−4^) prepared in sterile 0.1% peptone water. From each dilution, 100 μL was plated onto Slanetz–Bartley agar (Neogen, USA) and incubated at 41.5°C for 36–48 h. Colonies characteristic of enterococci (small red or maroon colonies) were selected (one isolate per sample was retained) and subcultured on nutrient agar at 37°C for 24 h to obtain pure cultures (Habib et al. 2022). Because only one representative colony was retained per positive sample, co‐colonising strains or minority resistant subpopulations may not have been detected.

All purified isolates (both presumptive *Salmonella* and *Enterococcus*) were then identified by MALDI‐TOF MS using the Autobio MS1000 system (Autobio Diagnostics, Zhengzhou, China). Briefly, single colonies were smeared onto the stainless‐steel target plate, overlaid with α‐cyano‐4‐hydroxycinnamic acid matrix and air‐dried. Spectra were acquired using the manufacturer's software, and identification scores ≥ 9.000 were considered reliable for species‐level assignment. Confirmed isolates were stored at −80°C for subsequent analyses (Park et al. 2021).

### 2.3 Phenotypic Antimicrobial Susceptibility Testing (AST)

AST for all confirmed *Salmonella* was performed using the VITEK 2 Compact automated system (bioMérieux, Marcy l'Étoile, France) in accordance with the manufacturer's instructions. The AST‐GN96 card was used, and minimum inhibitory concentration (MIC) values were generated automatically by the instrument software. This panel includes third‐ and fourth‐generation cephalosporins tested both alone and in combination with clavulanic acid, enabling phenotypic detection of extended‐spectrum β‐lactamase (ESBL) production based on clavulanate synergy. AST of *Salmonella* isolates was extended to 17 antimicrobial agents spanning veterinary‐relevant and clinically important antimicrobial classes used for AMR surveillance. The panel included beta‐lactams, including cephalosporins and imipenem; aminoglycosides; fluoroquinolones/quinolones; tetracycline; phenicols and folate pathway inhibitors. Some agents, such as imipenem, were included as sentinel compounds for surveillance and interpretive context rather than as routine therapeutic options in zoo ungulates.

Following species identification by MALDI‐TOF MS, only *E. faecium* and *Enterococcus faecalis* were selected for AST, as these species are the most clinically relevant enterococci in human and veterinary medicine and are commonly associated with multidrug resistance (MDR) and zoonotic transmission potential (Kim et al. 2024; Zaidi et al. 2024). For the selected clinically relevant *Enterococcus* isolates ( *E. faecium* and *E. faecalis* ), susceptibility testing was conducted using the VITEK 2 AST‐P592 card. This panel assessed activity against 10 antimicrobials: ampicillin, erythromycin, ciprofloxacin, high‐level gentamicin, high‐level streptomycin, tigecycline, teicoplanin, tetracycline, vancomycin and linezolid.

For *Salmonella*, *E. faecium* and *E. faecalis* MIC results were interpreted according to the Clinical and Laboratory Standards Institute (CLSI) performance standards (CLSI (Clinical and Laboratory Standards Institute) 2023). Isolates were categorised as MDR when nonsusceptibility was observed to at least one antimicrobial agent in three or more antimicrobial classes, following internationally accepted criteria.

### 2.4 Whole‐Genome Sequencing Workflow and in Silico Characterisation

Genomic DNA library preparation and Illumina sequencing (2 × 150 bp) were outsourced to Novogene (https://www.novogene.com/; Cambridge, UK) using an Illumina HiSeq platform (Illumina, San Diego, CA, USA). Sequence data were processed in batch using the Solu Genomics platform (Solu Healthcare Inc., Helsinki, Finland; v1.0.600) to support standardised assembly and downstream characterisation (Saratto et al. 2025). The analysis pipeline covered *de novo* assembly followed by species verification, molecular typing, detection of acquired AMR determinants and resistance‐associated mutations, plasmid reconstruction/typing and virulence gene screening (Saratto et al. 2025). Species assignments were independently validated using Kraken2 v2.1.3 against the PlusPF‐16 reference database (15 October 2025 release; https://github.com/DerrickWood/kraken2). Multilocus sequence types were determined with mlst v2.23.0 using allele and ST definitions from PubMLST (https://github.com/tseemann/mlst). Acquired AMR genes and relevant point mutations were identified using AMRFinderPlus v4.0.23–2025‐07‐16.1 under default settings (https://github.com/ncbi/amr). Plasmid content was characterised using MOB‐suite v3.1.9 (https://github.com/phac‐nml/mob‐suite). Virulence factors are screened using ABRicate v1.0.1 (https://github.com/tseemann/abricate) against the Virulence Factor Database (VFDB; https://www.mgc.ac.cn/VFs/). For *Salmonella* isolates, *in silico* serovar prediction was performed using SISTR v1.1.3 (Yoshida et al. 2016). Finally, the genomic sequences were evaluated for predicted pathogenic capacity in humans using the Global Pathogen Analysis Platform via PathogenFinder2, as a supplementary genome‐content screen (Ferrer Florensa et al. 2025). Raw sequencing reads were deposited in the National Center for Biotechnology Information (NCBI) under BioProject PRJNA1438090.

## 3 Results

### 3.1 Culture‐Based Detection of NTS and Enterococcus spp.

Results in Table 1 describe the species identity of the retained representative isolate per positive sample rather than the full within‐sample community. Across faecal samples from 101 captive ungulates in Al Ain Zoo, NTS was detected in 4/101 samples (3.9%, 95% CI 1.5%–9.7%), while *Enterococcus* spp. were detected in 77/101 samples (76.2%, 95% CI 67.1%–83.5%) (Table 1). The four *Salmonella* isolates comprised serovars Schwarzengrund (*n* = 2), Kentucky (*n* = 1) and Chester (*n* = 1) (Table 1). While the 77 *Enterococcus* isolates were spread over five species, of which *E. faecium* (*n* = 32) and *Enterococcus casseliflavus* were well represented, each accounted for 41.5% (32/77) of the overall *Enterococcus* examined (Table 1). Consistent with the study workflow, one isolate per sample was confirmed by MALDI‐TOF MS and species counts therefore equalled the number of positive samples (Table 1). The identified *E. faecium* (*n* = 32) and *E. faecalis* (*n* = 1) were included in the downstream AST, as these species are the most clinically relevant enterococci in human and veterinary medicine and are commonly associated with zoonotic transmission potential.

**TABLE 1:** Culture‐based detection of nontyphoidal *Salmonella enterica* and *Enterococcus* spp. in faecal samples from captive ungulates (*n* = 101) at Al Ain Zoo, United Arab Emirates.

Bacterial group	Positive/total (*n*/*N*)^a^	Detection%, 95% CI^b^	Serovars/species among isolates (*n*)
Nontyphoidal *Salmonella*	4/101	3.9% (1.5%–9.7%)	Serovars (*n*): Schwarzengrund (2); Kentucky (1); Chester (1)
*Enterococcus* spp.	77/101	76.2% (67.1%–83.5%)	Species (*n*): *Enterococcus faecium* (32); *Enterococcus casseliflavus* (32); *Enterococcus hirae* (9); *Enterococcus mundtii* (2); *Enterococcus avium* (1); *Enterococcus faecalis* (1)

^a^ One isolate per sample was identified by MALDI‐TOF MS; counts equal the number of positive samples.

^b^ 95% confidence interval calculated using the Wilson method.

### 3.2 Phenotypic Antimicrobial Susceptibility Profiles and MDR Classification

**FIGURE 1:**
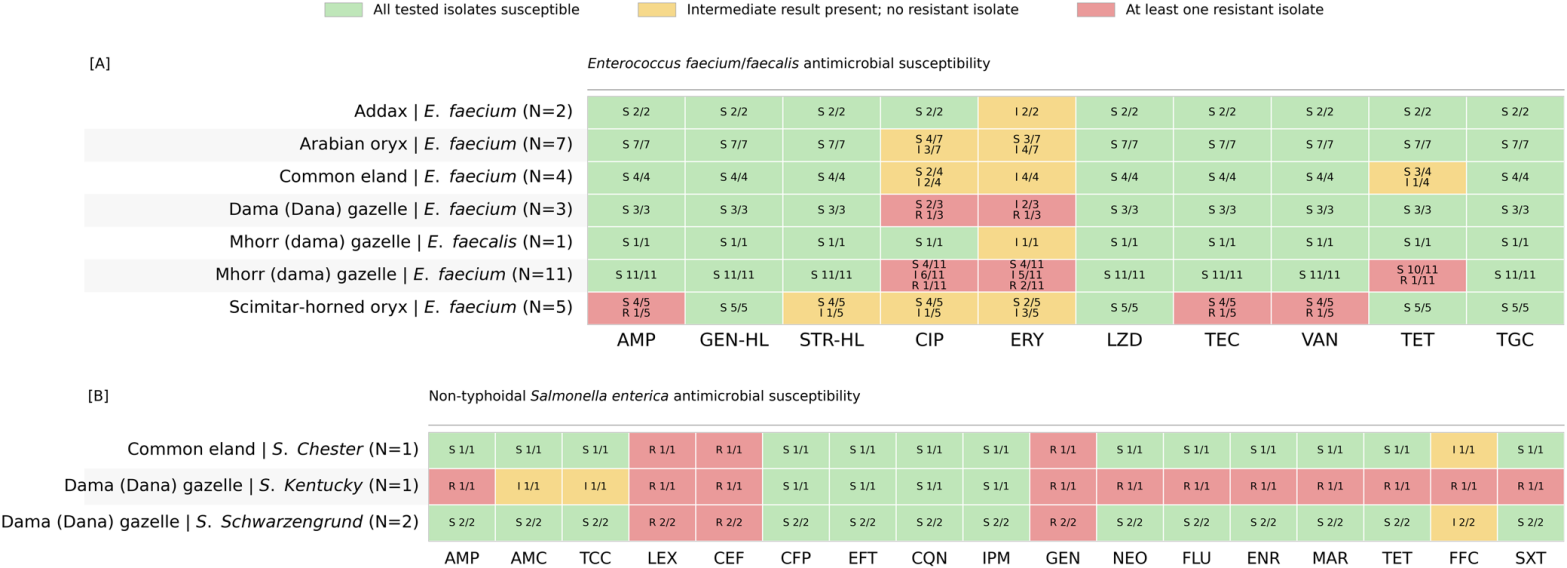
Heatmap of phenotypic antimicrobial susceptibility profiles of (A) *Enterococcus faecium* and *Enterococcus faecalis* isolates and (B) nontyphoidal *Salmonella enterica* isolates recovered from captive ungulates at Al Ain Zoo, United Arab Emirates. Cell values are shown as raw S/I/R counts, where S, susceptible; I, intermediate; *R*, resistant; for example, ‘S 2/2’ indicates that 2 of 2 isolates were susceptible. Row labels indicate the number of isolates tested for each host–bacterial group combination. AMC, amoxicillin–clavulanic acid; AMP, ampicillin; CEF, cefalotin; CFP, cefoperazone; CIP, ciprofloxacin; CQN, cefquinome; EFT, ceftiofur; ENR, enrofloxacin; ERY, erythromycin; FFC, florfenicol; FLU, flumequine; GEN, gentamicin; GEN‐HL, high‐level gentamicin; IPM, imipenem; LEX, cefalexin; LZD, linezolid; MAR, marbofloxacin; NEO, neomycin; STR‐HL, high‐level streptomycin; SXT, trimethoprim–sulfamethoxazole; TCC, ticarcillin–clavulanic acid; TEC, teicoplanin; TET, tetracycline; TGC, tigecycline; VAN, vancomycin.

Heatmap summaries are shown in Figure 1, where cell values are presented as S/I/R percentages (susceptible/intermediate/resistant; e.g., 100/0/0) for each animal–organism combination and antimicrobial. Phenotypic AST for *Enterococcus* focused on 33 isolates ( *E. faecium* , *n* = 32; *E. faecalis* , *n* = 1) recovered from six host groups (Mhorr (dama) gazelle, Arabian oryx, scimitar‐horned oryx, common eland, dama (Dana) gazelle and addax). Resistance was uncommon and concentrated in a limited subset of agents. Across the panel, the highest resistance frequency was observed for erythromycin (9.1%; 3/33) and ciprofloxacin (9.1%; 3/33). Resistance to ampicillin (3.0%; 1/33), tetracycline (3.0%; 1/33), vancomycin (3.0%; 1/33) and teicoplanin (3.0%; 1/33) was detected at low frequency, while 0% resistance was observed for linezolid, tigecycline and both high‐level aminoglycoside synergy screens (Figure 1).

Phenotypic AST was done for the four recovered NTS isolates (serovars Schwarzengrund, *n* = 2; Kentucky, *n* = 1; Chester, *n* = 1). A shared nonsusceptibility pattern was observed across all four isolates driven by uniform resistance to cefalexin (100%; 4/4), cefalotin (100%; 4/4) and gentamicin (100%; 4/4), together with florfenicol nonsusceptibility (100%; 4/4) (intermediate in 3/4 and resistant in 1/4) (Figure 1). Additional resistance was confined to a single isolate (serovar Kentucky), which displayed resistance to multiple antimicrobial classes including ampicillin, neomycin, flumequine, enrofloxacin, marbofloxacin, tetracycline, florfenicol and trimethoprim/sulfamethoxazole and intermediate results for amoxicillin/clavulanate and ticarcillin/clavulanate (Figure 1).

Applying the study MDR definition (resistance to > 1 agent in ≥ 3 antimicrobial groups), none (0/33) of the examined *Enterococcus* isolates met MDR criteria based on phenotypic AST. Nevertheless, one *E. faecium* isolate (from scimitar‐horned oryx) showed concurrent glycopeptide resistance (vancomycin and teicoplanin) alongside ampicillin resistance, and this isolate was retained for WGS as the phenotypically most clinically relevant profile within the *Enterococcus* dataset. On the other hand, 4/4 (100%) NTS isolates met MDR criteria, reflecting the combined nonsusceptibility across cephalosporins, aminoglycosides and phenicols in all isolates, with substantially broader class‐level nonsusceptibility in the serovar Kentucky isolate (Figure 1).

### 3.3 Whole‐Genome Sequencing Characterisation

WGS was performed for all NTS isolates and for a phenotypically prioritised *E. faecium* isolate (Table 2). The sequenced *E. faecium* isolate (ZEF) was assigned to ST22 and carried AMR determinants including *aac(6′)‐I* (aminoglycoside resistance), *msr(C)* (macrolide resistance) and a *liaR_E75K* substitution (associated with reduced susceptibility to daptomycin) (Table 2). Two plasmids were reconstructed, including a 50,719 bp plasmid (MOB cluster AD907, relaxase MOBP, conjugative) and a 23,029 bp plasmid (MOB cluster AB759, relaxase MOBP, mobilisable) (Table 2).

**TABLE 2:** Whole‐genome sequencing based characterisation of all nontyphoidal *Salmonella enterica* isolates and a multidrug‐resistant *Enterococcus faecium* from captive ungulates at Al Ain Zoo, United Arab Emirates.

Isolate‐serovar/species	Short‐read quality	Sequence type	Antimicrobial resistance determinants	Plasmids			
				Size (bp)	MOB cluster	Relaxase types	Putative mobility
ZEF‐ *E. faecium*	Mean read length: 149.9 bp	ST22	*aac(6′)‐I, liaR_E75K, msr(C)*	50,719	AD907	MOBP	Conjugative
				23,029	AB759	MOBP	Mobilisable
	Coverage: 665×						
	N50: 231 kbp						
ZS1‐*Salmonella* serovar Schwarzengrund	Mean read length: 150.0 bp	ST241	—^a^	54,608	AC578	MOBF	Conjugative
	Coverage: 356×						
	N50: 337 kbp						
ZS2‐*Salmonella* serovar Kentucky	Mean read length: 150.0 bp	ST198	*aadA1, aadA2, aph(3′)‐Ia, bla*_TEM‐57_, *dfrA12, sul1, sul3, tet(A), cmlA1, qacEdelta1, qacL, parC_S80I, gyrA_D87G, gyrA_S83F*	21,304	AC247	—	Nonmobilisable
	Coverage: 520×		
	10,841			AG658	—	Nonmobilisable
	N50: 645 kbp		
				5865	AA525	MOBP	Mobilisable
4345		AA575	MOBQ	Mobilisable
4221	AA579	MOBQ	Mobilisable
ZS3‐*Salmonella* serovar Schwarzengrund	Mean read length: 150.0 bp	ST241	—	54,608	AC578	MOBF	Conjugative
	Coverage: 307×						
	N50: 425 kbp						
ZS4‐*Salmonella* serovar Chester	Mean read length: 150.0 bp	ST2063	—	—	—	—	—
	Coverage: 404×						
	N50: 587 kbp						

^a^ None identified.

For NTS, both Schwarzengrund isolates (ZS1 and ZS3) belonged to ST241, and each carried a 54,608 bp conjugative plasmid (MOB cluster AC578, relaxase MOBF) (Table 2). The Kentucky isolate (ZS2) was ST198 and harboured a broad resistome including aminoglycoside, β‐lactam, sulfonamide/trimethoprim, tetracycline, phenicol and quinolone resistance‐associated determinants and mutations, and quaternary ammonium (disinfectant)‐associated genes (Table 2). Multiple plasmids were reconstructed for ZS2, including two nonmobilisable plasmids and three mobilisable plasmids (Table 2). The Chester isolate (ZS4) was assigned to ST2063 and did not show plasmid reconstruction entries (Table 2).

### 3.4 Virulence Gene Content and PathogenFinder‐Predicted Human Pathogenic Capacity

**FIGURE 2:**
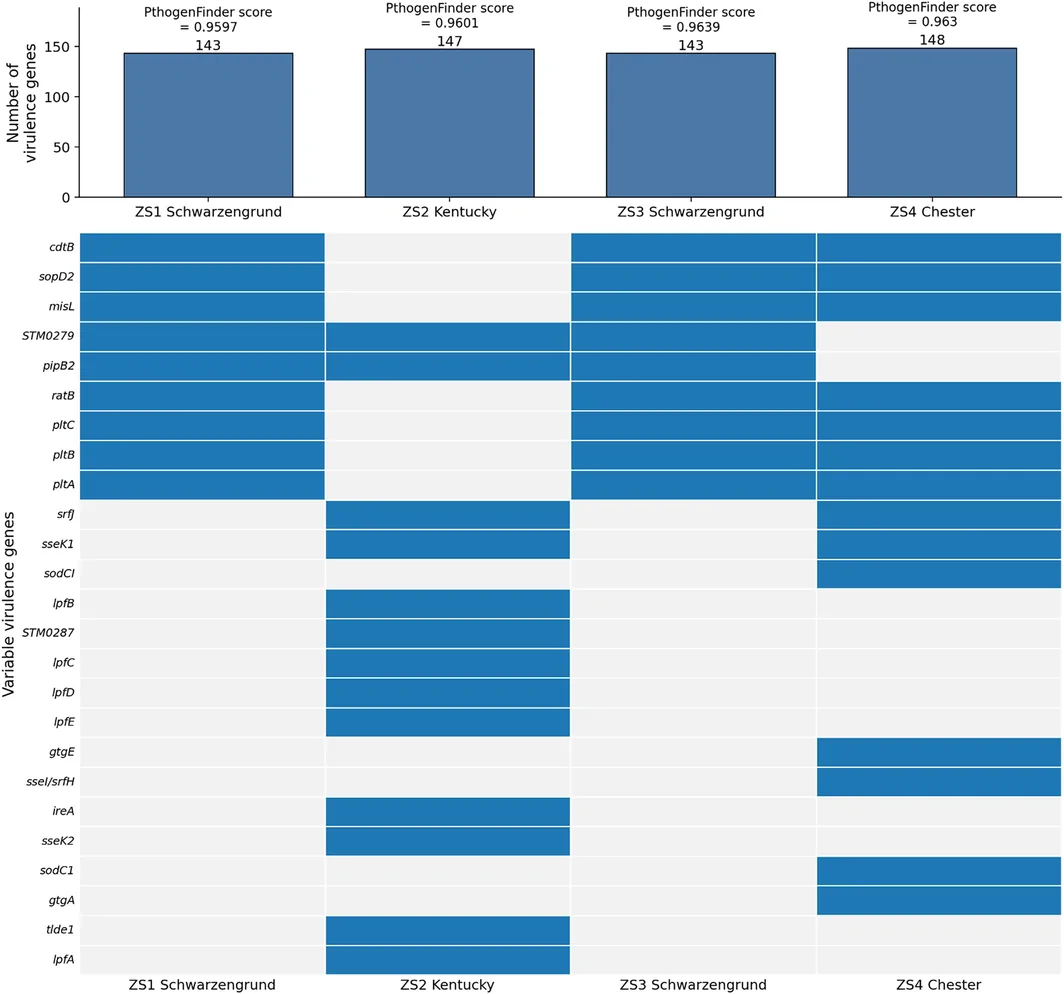
Virulence gene content and predicted human pathogenic potential of four nontyphoidal *Salmonella* isolates recovered from captive ungulates at Al Ain Zoo, United Arab Emirates. The upper panel shows the total number of virulence‐associated genes detected per isolate, with the PathogenFinder score (predicted human pathogenic capacity) displayed above each bar. The lower panel presents a binary heatmap of the distribution of variable virulence genes across isolates, indicating presence (blue) or absence (white) across isolates to highlight differences in gene repertoires.

Across the four NTS genomes, the number of detected virulence‐associated genes ranged from 143 to 148 (Figure 2). PathogenFinder scores were consistently high (0.9597 for ZS1 Schwarzengrund; 0.9601 for ZS2 Kentucky; 0.9639 for ZS3 Schwarzengrund; 0.963 for ZS4 Chester), all exceeding the > 0.5 threshold used to indicate predicted human pathogenic capacity (Figure 2). Comparative profiling identified a set of variable virulence genes that differed among isolates (Figure 2). Across the four NTS isolates, 26 virulence genes were variable (present in a subset rather than all genomes), with the Kentucky and Chester genomes showing a larger share of the variable repertoire than the two Schwarzengrund genomes (Figure 2).

For the sequenced *E. faecium* isolate, virulence screening identified only two virulence‐associated genes (*acm* and *scm*; collagen adhesin/binding precursors) (Table S1). The PathogenFinder score for this *E. faecium* genome was 0.6138 (0.0441).

Functional annotation of virulence genes shared (*n* = 134) among the NTS genomes indicated representation across adhesion/colonisation and secretion‐associated categories, including fimbrial colonisation (nine genes), curli/biofilm‐associated determinants (eight genes), SPI‐1 structural components (21 genes) and effectors/regulators (14 genes), SPI‐2 secretion apparatus genes (22 genes) and intracellular survival effectors (10 genes), global regulators (seven genes), iron acquisition (two genes) and additional conserved virulence‐associated genes (41 genes) (Table S1). For *Enterococcus*, the virulence complements in the sequenced isolate comprised *acm* and *scm* (two genes), categorised as collagen adhesin/binding precursors (Table S1).

## 4 Discussion

AMR is increasingly recognised as a One Health problem shaped by interactions across human, animal and environmental systems (Larsson et al. 2023). In the Middle East, conservation‐managed ungulates are central to biodiversity, and zoos provide a managed setting where animal health, husbandry and environmental hygiene intersect (Low Cunningham 2004). In the studied urban zoological environment in the UAE, we detected NTS in a small proportion of clinically healthy ungulates alongside frequent carriage of *Enterococcus* spp. All NTS isolates met the study MDR definition, and the *Salmonella* serovar Kentucky isolate showed the broadest nonsusceptibility and resistome. While phenotypic resistance among *Enterococcus* isolates was generally uncommon, we identified a glycopeptide‐resistant *E. faecium* isolate with a daptomycin‐associated genomic determinant. WGS resolved serovar/species composition, plasmid architectures and predicted high human‐pathogenic potential for the NTS genomes, providing a regional baseline for conservation‐priority zoo ungulates.

Within the examined ungulates in the present study setting, low NTS detection alongside high *Enterococcus* recovery was consistent with the contrasting ecology of these taxa: NTS carriage can be intermittent and exposure‐driven, whereas enterococci are common commensals that persist under diverse conditions (Rukambile et al. 2019). Subclinical *Salmonella* carriage has been reported in other zoo collections, including a Mexican survey documenting diverse nontyphoidal serovars (Silva‐Hidalgo et al. 2012) and a South African national zoo study detecting *Salmonella* in captive animals (Mtshali 2012). Similar faecal surveys in free‐roaming wildlife in South Korea have also detected *Salmonella* (Mafizur et al. 2024). Nevertheless, the most consequential signal in our dataset was MDR in all NTS isolates recovered from asymptomatic hosts. In conservation‐managed herds, such resistance can narrow therapeutic options if clinical salmonellosis occurs and can increase the operational consequences of outbreaks (Marin et al. 2022). The identification of *Salmonella* serovar Kentucky ST198 was noteworthy because this lineage has been linked to international dissemination of MDR strains and high‐level fluoroquinolone resistance in human surveillance (Mohamed et al. 2025). Although we did not infer sources or transmission, the broader resistome in our *Salmonella* ST198 isolate aligned with this high‐concern lineage (Le Hello et al. 2013).

The *Enterococcus* findings complemented the NTS results by reflecting commensal carriage in the same managed environment. Enterococci are used as indicators of faecal contamination and antimicrobial selection because they are abundant, resilient and genetically adaptable (Zaidi et al. 2024). Resistance profiles in wildlife and captivity have varied, including reports from a zoo collection (Kim et al. 2024), captive parrots (de Freitas et al. 2018) and free‐living primates (Grassotti et al. 2018). Genomic work in free‐living birds supports environmental reservoirs for potentially pathogenic *Enterococcus* lineages (Kwit et al. 2023). In our data, no *E. faecium* / *E. faecalis* isolates met the phenotypic MDR definition, but a single glycopeptide‐resistant *E. faecium* isolate was clinically notable, based on its phenotypic resistance to priority last‐line antimicrobials (glycopeptides) (van Groesen et al. 2022). WGS assigned it to ST22, which has been identified before in *E. faecium* from healthy humans and free‐living birds (Kwit et al. 2023; Tedim et al. 2024). Additionally, WGS identified an amino‐acid substitution in *liaR_E75K* associated with reduced susceptibility to daptomycin, a last‐line agent used against severe MDR Gram‐positive infections (Zeng et al. 2022). Because daptomycin was not included in the phenotypic AST panel, the *liaR_E75K* substitution should be interpreted only as a genomic alert requiring confirmatory daptomycin MIC testing. Similarly, the glycopeptide phenotype should be confirmed by reference MIC testing and targeted evaluation of known glycopeptide‐resistance determinants before mechanistic interpretation. Such findings underscore the importance of integrating genomic analyses with phenotypic testing to better interpret AMR mechanisms and their potential clinical and ecological implications.

WGS is increasingly accepted as a gold‐standard approach for *Salmonella* serovar determination, offering higher resolution and overcoming several limitations associated with conventional serotyping methods (Yoshida et al. 2016). In the present study in a zoo setting, WGS added resolution beyond AST by placing each NTS isolate within recognised serovar and sequence types and characterising mobile genetic elements. The two isolates belonging to *Salmonella* serovar Schwarzengrund carried a conjugative plasmid and were assigned ST241 that has been described in poultry‐associated *Salmonella* Schwarzengrund with evidence of dissemination in broiler systems (Felix et al. 2025). The serovar Chester isolate was ST2063, a sequence type reported in *Salmonella* from geographically structured clusters and was associated with travel‐associated salmonellosis outbreaks (Fonteneau et al. 2017; Siira et al. 2019). These contextual reports do not imply direct linkage to our zoo setting but illustrate how WGS enables standardised comparisons across sectors.

Virulence profiling and PathogenFinder predictions further supported One Health relevance of the present study findings. All NTS genomes carried large virulence gene repertoires with conserved SPI‐1/SPI‐2 machinery, and PathogenFinder scores were uniformly high (> 0.95), consistent with predicted human‐pathogenic potential based on genome content (Ferrer Florensa et al. 2025). Such predictions should be interpreted as compatibility with human pathogenicity rather than evidence of transmission. For the sequenced *E. faecium* , only the collagen adhesin/binding genes *acm* and *scm* were detected, and the PathogenFinder score was modestly above the 0.5 threshold (0.6138 [0.0441]) (Ferrer Florensa et al. 2025). Overall, these findings reinforced the urban zoo as a sentinel environment at the human–animal–environment interface. Culture‐based detection, standardised AST and WGS offered complementary information for zoo biosecurity by identifying subclinical carriages in conservation animals and defining MDR and mobile‐element features relevant to management.

Several limitations should be noted. The study was cross‐sectional and limited to a single institution, restricting inference about temporal dynamics or persistence. The small number of NTS isolates constrained epidemiological conclusions and host‐level comparisons. The *Enterococcus* genomic component was limited to one phenotypically prioritised *E. faecium* isolate and therefore cannot support conclusions about the genomic diversity or resistome of *Enterococcus* spp. in the sampled population. Environmental, feed, water and human sampling were not included, preventing possible source attribution. Finally, the one‐isolate‐per‐sample approach improved comparability but almost certainly reduced detection of within‐sample diversity, mixed serovars/species and co‐occurring resistance profiles. Despite these limitations, this study represents the first investigation in the UAE (and to our knowledge the wider Middle East) focusing on AMR and genomic characterisation of these specific groups of enteric bacteria in native conservation‐priority ungulates. The study was conducted within one of the region's leading zoological institutions for wildlife conservation; as such, the findings provide an important baseline for future surveillance and One Health research in zoological and conservation settings.

## 5 Conclusion

In conclusion, conservation‐priority ungulates housed in an urban UAE zoo carried both NTS and *Enterococcus* spp., including MDR NTS lineages and a phenotypically notable *E. faecium* isolate with glycopeptide nonsusceptibility that warrants confirmatory testing and further genomic investigation. These findings support the usefulness of targeted zoo‐based faecal surveillance as one component of One Health AMR monitoring, while broader longitudinal sampling of animals, environment, feed, water, staff‐contact points and visitors would be required to evaluate transmission pathways.

## Author Contributions

**Ihab Habib:** conceptualization, methodology, formal analysis, supervision, writing – original draft, writing – review and editing, funding acquisition. **Moneeb A. Qablan:** data curation, supervision, validation, resources, writing – review and editing. **Fatema Aaref Alzaabi:** writing – review and editing, investigation, validation, formal analysis. **Fatema Rashed Alnuaimi:** investigation, validation, formal analysis, writing – review and editing. **Ghalya Ahmed Almemari:** investigation, validation, formal analysis, writing – review and editing. **Glindya Bhagya Lakshmi:** supervision, project administration, validation, methodology, writing – review and editing. **Safeya Al Naqbi:** data curation, resources, validation, writing – review and editing. **Fatima Al Shamsi:** data curation, resources, validation, writing – review and editing. **Dhanya Vijay:** data curation, resources, validation, writing – review and editing. **John Mhongovoyo:** resources, project administration, validation, writing – review and editing. **Sultan Khalifa Alkaabi:** resources, project administration, validation, writing – review and editing. **Amna Mana Alotaiba:** resources, project administration, validation, writing – review and editing, funding acquisition.

## Funding

This work was supported by the United Arab Emirates University (G00005128, G00005130).

## Ethics Statement

No animal ethics approval was required, as faecal samples were collected noninvasively during routine husbandry without animal handling or experimental intervention.

## Conflicts of Interest

The authors declare no conflicts of interest.

## Supporting information


**Table S1:** Functional categorisation of the virulence genes shared among the genomes of nontyphoidal *Salmonella* isolates, and multidrug‐resistant *Enterococcus faecium* from captive ungulates at Al Ain Zoo, United Arab Emirates.

## Data Availability

The sequencing data and assembled genomes generated in this study have been publicly deposited in the NCBI database under BioProject accession number PRJNA1438090.
