# Supplementary material for: Antimicrobial Resistance in Nontyphoidal Salmonella and Clinically Relevant Enterococcus From Faecal Samples of Conservation‐Priority Captive Ungulates in a United Arab Emirates Urban Zoo: A Cross‐Sectional Baseline Study
Source: Environ Microbiol Rep. 2026 Sep 16;18(5):e70416. doi: 10.1111/1758-2229.70416 (PMC13583175; doi:10.1111/1758-2229.70416)
Supplement: Supplementary file 1 [file EMI4-18-e70416-s001.docx]

**Supplementary Table 1**

Functional categorization of the virulence genes shared among the genomes of non-typhoidal *Salmonella* isolates, and multidrug-resistant *Enterococcus faecium* from captive ungulates at Al Ain Zoo, United Arab Emirates

| **Bacterial group** | **Category** | **Gene count** | **Genes** | **Biological role** |
| --- | --- | --- | --- | --- |
| Non-typhoidal *Salmonella* | Adhesion and fimbrial colonization | 9 | *fimA, fimC, fimD, fimF, fimH, fimI, fimW, fimY, fimZ* | Mediates host tissue attachment, intestinal colonization, and persistence through fimbrial adhesins. |
|  | Curli and biofilm formation | 8 | *bapA, csgA, csgB, csgC, csgD, csgE, csgF, csgG* | Promotes surface attachment, multicellular behavior, environmental persistence, and biofilm development. |
|  | Salmonella Pathogenicity Island 1 structural apparatus | 21 | *invA, invB, invC/sctN, invE, invF, invG, invH, invI, invJ, orgA/sctK, orgB/SctL, orgC, prgH, prgI, prgJ, prgK, spaO/sctQ, spaP, spaQ, spaR, spaS* | Encodes the structural components of the type three secretion system required for epithelial cell invasion. |
|  | Salmonella Pathogenicity Island 1 effectors and invasion associated regulators | 14 | *avrA, hilA, hilC, hilD, iacP, iagB, sipA/sspA, sipB/sspB, sipC/sspC, sipD, sopA, sopB/sigD, sopD, sopE2* | Includes invasion effectors and regulatory factors that coordinate host cell entry and early infection events. |
|  | Salmonella Pathogenicity Island 2 secretion apparatus | 22 | *ssaC, ssaD, ssaE, ssaG, ssaH, ssaI, ssaJ, ssaK, ssaL, ssaM, ssaN, ssaO, ssaP, ssaQ, ssaR, ssaS, ssaT, ssaU, ssaV, ssaX, sscA, sscB* | Encodes the type three secretion machinery required for intracellular survival and systemic infection. |
|  | Salmonella Pathogenicity Island 2 effectors and intracellular survival | 10 | *sifA, sseA, sseB, sseC, sseD, sseE, sseF, sseG, sseJ, sseL* | Includes secreted effectors that remodel host cells and promote survival within *Salmonella*-containing vacuoles. |
|  | Global virulence regulators and signaling | 7 | *fur, phoP, phoQ, pmrA, pmrB, sprB, ssrA* | Controls virulence gene expression, stress adaptation, antimicrobial resistance responses, and host adaptation. |
|  | Iron acquisition and metal homeostasis | 2 | *iroB, iroC* | Supports acquisition and regulation of essential metals needed for growth and infection in the host. |
|  | Other conserved virulence-associated genes | 41 | *STM0266, STM0267, STM0268, STM0269, STM0270, STM0271, STM0272, STM0273, STM0274, STM0275, STM0276, STM0278, STM0282, STM0283, STM0284, STM0285, STM0286, STM0290, mgtB, mgtC, mig-14, pagN, pipB, rpoS, sicA, sicP, sifB, siiE, sinH, slrP, spiC/ssaB, sptP, ssrB, steA, steB, steC, steD, tae4, tssJ, tssK, vgrG* | Contains additional conserved virulence-related genes involved in colonization, intracellular fitness, secretion-associated functions, and stress adaptation. |
| *Enterococcus* spp. | Collagen adhesin/binding precursor | 2 | *acm, scm* | Promote attachment to host extracellular matrix and tissues, supporting colonization and persistence (and are implicated in endocarditis-associated adherence). |
